# Influence of life expectancy on shared decision-making for prostate cancer screening

**DOI:** 10.1007/s10552-025-02069-1

**Published:** 2025-09-10

**Authors:** Myles M. Reed, Jialin Mao, Meenakshi Davuluri, Neal A. Patel, Bashir Al Hussein Al Awamlh, Kevin H. Kensler

**Affiliations:** 1 MD Program, Weill Cornell Medical College, New York, NY USA; 2 Department of Population Health Sciences, Weill Cornell Medicine, New York, NY USA; 3 Sandra and Edward Meyer Cancer Center, Weill Cornell Medicine, 425 E 61st St, DV-306, New York, NY USA; 4 Department of Urology, Weill Cornell Medicine/NewYork-Presbyterian, New York, NY USA

**Keywords:** Prostate cancer, Cancer screening, Shared decision-making, Prostate-specific antigen, Life expectancy

## Abstract

**Purpose:**

The U.S. Preventive Services Task Force recommends that men aged 55–69 years undergo shared decision-making (SDM) regarding prostate cancer (PCa) screening, and routine screening is not recommended for older men or those with limited life expectancy. We evaluated the association between individual life expectancy and the receipt and content of SDM.

**Methods:**

We identified male respondents aged ≥ 50 years and older without a history of PCa within the 2020 Behavioral Risk Factor Surveillance System survey. SDM was defined as whether a healthcare professional had ever discussed the advantages and/or disadvantages of a prostate-specific antigen test with the respondent. Life expectancy was estimated based on a validated index incorporating respondent age, comorbidities, and activities of daily living. Adjusted odds ratios (aOR) were estimated for the associations between respondent age, life expectancy, and receipt of SDM, accounting for healthcare access and utilization-related factors.

**Results:**

Reported receipt of any SDM was highest among 70–74-year-old men (62.6%, 95% CI 60.5–64.6%). In multivariable models, those with the lowest life expectancy were modestly less likely to receive SDM (aOR = 0.84, 95% CI 0.71–0.99, Q4 vs Q1) compared to those with the greatest life expectancy. SDM discussing only the advantages of screening was the most common form of SDM; the content of SDM conversations did not differ by age or life expectancy, and 14.5% of men who were recently screened reported no prior SDM whatsoever.

**Conclusions:**

Life expectancy appears to be inadequately incorporated into decision-making regarding PCa screening. Additional tools are needed to support SDM conversations to improve the population benefits–harms of PCa screening.

**Supplementary Information:**

The online version contains supplementary material available at 10.1007/s10552-025-02069-1.

## Introduction

The potential benefits and harms of prostate cancer (PCa) screening vary by age and health status. Older males or those with limited life expectancy due to comorbidity may not accrue a mortality benefit from screening given the long natural history of prostate cancer and face an increased risk of overdiagnosis of indolent tumors [1–3]. Given uncertainty about the relative benefits and harms of routine screening, the U.S. Preventive Services Task Force (USPSTF) and other national guidelines recommend that males at average risk should undergo shared decision-making (SDM) about prostate-specific antigen (PSA)-based screening with a healthcare provider starting at as early as 45 years of age [4–7]. Per national guidelines, routine screening is not recommended for males 70 years or older or for those with less than 10 years of life expectancy [4–7]. Despite these recommendations, SDM regarding prostate cancer and screening rates remain high after age 70, and lower life expectancy is only modestly associated with lower screening rates [8–11]. Although several determinants of SDM have been identified previously, the association between estimated life expectancy and SDM, and subsequent PCa screening remains unclear [12–14].

SDM for prostate cancer screening using PSA should involve a conversation between patients and healthcare providers that discusses the potential benefits and the harms of PCa screening and incorporates patient preferences and values [15]. Effective SDM can enhance communication and trust between patients and providers, clarify risks and benefits of procedures, empower patients, and align care received with patient values [15]. As these benefits and harms inherently vary based on patient health status, life expectancy should be a significant consideration in SDM conversations. Given the central role of SDM in prostate cancer screening recommendations, we used data from a national survey to examine how patterns in the receipt of SDM and the content of SDM conversations varied by patient age and life expectancy. We further examined differences in prostate cancer screening rates with respect to patient age, life expectancy, and the content of SDM conversations.

## Materials and methods

### Behavioral Risk Factor Surveillance System

The Behavioral Risk Factor Surveillance System (BRFSS) is a system of landline and cellular telephone-based surveys conducted by state health departments and coordinated by the Centers for Disease Control and Prevention that query respondents about their health-related risk behaviors, chronic health conditions, and the use of preventive healthcare services [16]. The BRFSS employs a complex sampling frame such that results from the BRFSS are generalizable to the community-dwelling U.S. population. The BRFSS is conducted annually and asks respondents to report their cancer screening history in select survey years. The 2020 BRFSS is the most recent survey year in which a history of prostate cancer screening and shared decision-making regarding prostate cancer screening were ascertained nationwide. Response rates for the 2020 BRFSS varied across states and territories and ranged from 34.5% to 67.2% (median 47.9%) [16].

### Assessment of shared decision-making and prostate cancer screening

BRFSS respondents assigned male at birth and ages 40 or older at the time of the survey were asked in two separate items whether a doctor, nurse, or other health professional has ever talked with them about the advantages of the PSA test, and whether a doctor, nurse, or other health professional has ever talked with them about the disadvantages of the PSA test. Respondents who provided an affirmative response to either item were categorized as having ever undergone any SDM. Responses to these two items were also combined to assess whether respondents had assessed both the advantages and disadvantages, only the advantages, only the disadvantages, or neither the advantages nor disadvantages of the PSA test. Respondents who reported discussing both the advantages and disadvantages of the PSA test were categorized as having undergone complete SDM. BRFSS respondents also were asked whether they had ever received a PSA test, the reason for this test, and how long ago they received this test. Consistent with prior reports, we identified respondents who reported receiving a screening PSA test in the prior two years [8]. Respondents with an unknown prostate cancer screening or SDM history were excluded from the analytic subpopulation.

### Estimation of life expectancy

To estimate respondent 10-year mortality risk, we implemented the index developed by Cruz et al. in the Health and Retirement Study and adapted for use in the BRFSS by Moss et al. [17, 18]. This index incorporates respondent age, sex, body mass index, history of diabetes, history of cancer, history of lung disease, history of heart failure, smoking status, and difficulty dressing/bathing, conducting errands alone, and walking/climbing stairs. The index has been validated for individuals 50 years of age and older. Scores for the adapted mortality index for males ages 50 or older range from 2.5 to 24.5. The mortality risk score was categorized into quartiles based on the distribution of the index among male BRFSS respondents aged 50 years or older (3.5 or less, 4–6.5, 7–8.5, 9 or greater) and was used to represent estimated life expectancy. These quartiles correspond to predicted 10-year mortality risk of < 14%, 14–39%, 40–61%, and 62% or greater, respectively [17].

### Inclusion and exclusion criteria

Within the 2020 BRFSS, we identified a subpopulation of respondents who were assigned male at birth and were aged 50 years or greater at the time of the survey. National guidelines are heterogeneous with respect to the age at which men at average risk of PCa are recommended to start SDM, with the American Urological Association having the earliest starting age across organizations at 45 years [4–7]. However, we did not include participants aged 45–49 years as our life expectancy calculator was not validated in this age group, and thus restricted our analytic population to males aged 50 and older. Furthermore, respondents who reported a prior history of prostate cancer, had an unknown history of prostate cancer screening, or had received a prior non-screening PSA test were excluded from the final subpopulation of interest.

### Statistical analyses

In primary analyses, the proportions of screening-eligible respondents who underwent SDM for prostate cancer screening and the content of SDM discussions were stratified by respondent age and mortality risk score. In secondary analyses, these proportions were also estimated among those who reported and did not report undergoing screening in the prior two years. The associations between age and mortality risk score and receipt of any SDM or complete SDM were evaluated both in crude (containing only age and the mortality risk score) and multivariable logistic regression models, which additionally accounted for factors influencing healthcare access and utilization, including race and ethnicity, household income, educational attainment, employment status, health insurance status, marital status, and whether the respondent had a usual source of healthcare. These models containing both age and mortality risk score are hereafter described as mutually adjusted models. Heterogeneity in the association between the mortality risk score and receipt of SDM was evaluated through a likelihood ratio test for the inclusion of product terms between age categories and the continuous mortality risk score. The associations between age, mortality risk score, SDM, and receipt of a screening PSA test in the prior two years were also evaluated using multivariable logistic regression models that corrected for the factors listed above. In sensitivity analyses, we examined trends in SDM by ever receipt of a screening PSA test as opposed to within two years, which yielded consistent findings (results not shown). All statistical tests were two-sided with *p* values < 0.05 considered statistically significant. All analyses were performed in SAS (Version 9.4) and R (Version 4.4.1) using appropriate procedures to account for the BRFSS survey weights.

## Results

Among the 401,958 respondents to the 2020 BRFSS, 83,052 met criteria for inclusion in the analytic subpopulation (Supplemental Fig. 1). Respondent characteristics by mortality risk score are shown in Table 1. Respondents with lower life expectancy were older at the time of the survey. Non-Hispanic White respondents were more likely to have lower life expectancy than Hispanic/Latinx, Non-Hispanic American Indian/Alaska Native, and Non-Hispanic Asian or Pacific Islander respondents. Educational attainment was positively associated with mortality risk, while there was an inverse association for annual income. Retired respondents and respondents who were divorced, widowed, or separated have lower life expectancy. Individuals with health insurance or a usual source of healthcare had higher mortality risk. Respondent characteristics by receipt of any SDM regarding prostate cancer screening are shown in Supplemental Table 1. 59.2% of respondents who reported prior SDM underwent prostate cancer screening in the prior two years versus 9.6% of respondents who reported no prior SDM.

**Table 1:** Weighted frequencies of respondent characteristics by mortality risk score quartile in the 2020 Behavioral Risk Factor Surveillance System

Characteristic	Q1		Q2		Q3		Q4	
	Weighted n	%	Weighted n	%	Weighted n	%	Weighted n	%
Age at Survey								
50–54	5,923,531	42.1	2,029,344	14.1	482,792	7.5	322,845	3.9
55–59	4,836,762	34.4	2,170,973	15.1	394,803	6.1	408,926	5.0
60–64	3,302,142	23.5	3,507,136	24.4	823,594	12.8	778,650	9.4
65–69	–	–	4,374,145	30.5	1,079,706	16.8	858,180	10.4
70–74	–	–	1,416,919	9.9	1,829,830	28.5	1,847,635	22.4
75–79	–	–	861,480	6.0	1,162,942	18.1	1,411,564	17.1
80 +	–	–	–	–	656,154	10.2	2,617,819	31.7
Race and Ethnicity								
Hispanic/Latinx	2,091,344	14.9	1,761,558	12.3	685,418	10.7	879,779	10.7
Non-Hispanic American Indian/Alaska Native	94,647	0.7	147,732	1.0	65,464	1.0	92,769	1.1
Non-Hispanic Asian or Pacific Islander	695,427	4.9	568,407	4.0	201,117	3.1	181,264	2.2
Non-Hispanic Black	1,534,045	10.9	1,436,344	10.0	691,207	10.8	849,245	10.3
Non-Hispanic Multiracial	105,252	0.7	114,321	0.8	82,783	1.3	69,622	0.8
Non-Hispanic White	9,216,497	65.5	9,995,628	69.6	4,530,340	70.5	5,958,590	72.3
Other or Unknown	325,224	2.3	336,006	2.3	173,492	2.7	214,350	2.6
Annual Household Income								
< $25,000	1,362,749	9.7	2,659,460	18.5	1,578,280	24.5	2,658,003	32.2
< $50,000	1,754,882	12.5	2,609,394	18.2	1,399,461	21.8	1,867,084	22.6
< $75,000	1,747,758	12.4	2,037,822	14.2	851,117	13.2	1,005,405	12.2
$75,000 or more	7,484,498	53.2	5,029,145	35.0	1,548,921	24.1	1,314,018	15.9
Unknown	1,712,549	12.2	2,024,177	14.1	1,052,042	16.4	1,401,108	17.0
Educational Attainment								
Less than High School	1,169,280	8.3	1,965,057	13.7	1,009,500	15.7	1,825,016	22.1
High School or equivalent	3,510,116	25.0	3,940,925	27.4	1,807,258	28.1	2,409,157	29.2
Some College	3,947,013	28.1	4,257,364	29.6	1,848,865	28.8	2,278,417	27.6
College Graduate	5,388,045	38.3	4,174,440	29.1	1,743,224	27.1	1,698,209	20.6
Other	47,981	0.3	22,211	0.2	20,975	0.3	34,819	0.4
Employment Status								
Employed	11,327,387	80.6	7,010,536	48.8	1,541,646	24.0	868,353	10.5
Unemployed	851,623	6.1	910,404	6.3	233,443	3.6	228,108	2.8
Student/Homemaker/Unable to Work	406,448	2.9	1,117,246	7.8	805,977	12.5	1,340,624	16.3
Retired	1,336,250	9.5	5,202,753	36.2	3,812,526	59.3	5,765,881	69.9
Unknown	140,728	1.0	119,058	0.8	36,228	0.6	42,653	0.5
Health Insurance Status								
None	1,465,726	10.4	1,322,149	9.2	341,453	5.3	402,025	4.9
Insured	12,555,064	89.3	12,969,251	90.3	6,068,472	94.4	7,819,977	94.8
Unknown	41,645	0.3	68,597	0.5	19,896	0.3	23,617	0.3
Marital Status								
Married/Domestic Partner	10,489,761	74.6	9,862,004	68.7	4,195,869	65.3	4,608,347	55.9
Divorced/Widowed/Separated	2,207,447	15.7	3,169,510	22.1	1,744,118	27.1	3,042,964	36.9
Never Married	1,307,591	9.3	1,274,040	8.9	467,839	7.3	577,748	7.0
Unknown	57,636	0.4	54,443	0.4	21,995	0.3	16,561	0.2
Has Usual Source of Healthcare								
Yes	11,457,575	81.5	12,163,558	84.7	5,739,042	89.3	7,542,883	91.5
No	2,534,379	18.0	2,115,662	14.7	643,755	10.0	664,880	8.1
Unknown	70,481	0.5	80,777	0.6	47,024	0.7	37,856	0.5
Received Screening PSA Test in Prior Two Years								
Yes	4,251,006	30.2	5,423,278	37.8	2,409,865	37.5	2,518,719	30.5
No	9,811,429	69.8	8,936,719	62.2	4,019,956	62.5	5,726,900	69.5
Received Any SDM								
Yes	6,202,315	44.1	7,443,636	51.8	3,350,263	52.1	4,091,339	49.6
No	7,860,120	55.9	6,916,361	48.2	3,079,558	47.9	4,154,280	50.4

No men ages 70 or older were included in the lowest mortality risk quartile and no men ages 80 or older were included in the second mortality risk quartile

**Fig. 1:**
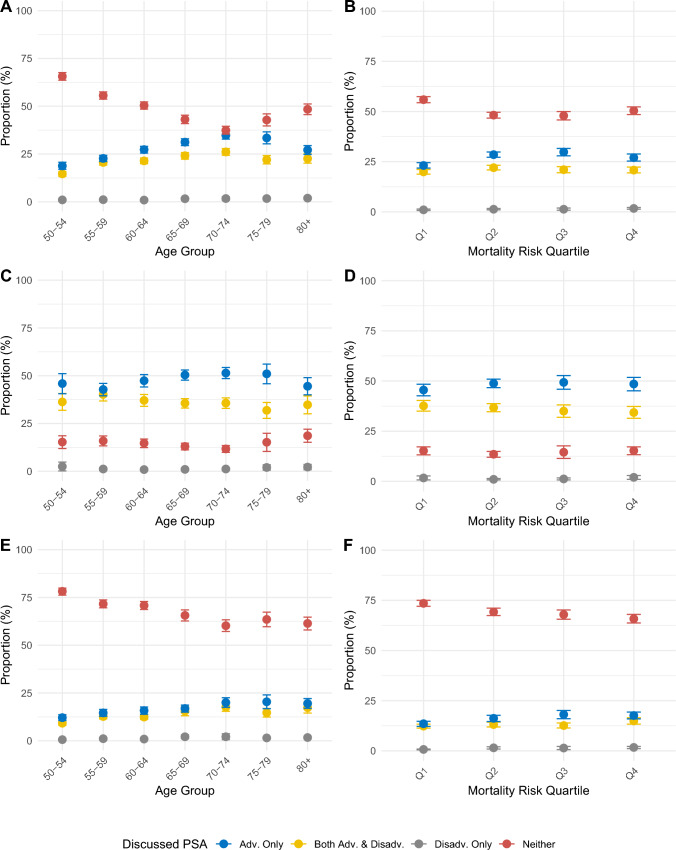
Proportions of men who report that a doctor, nurse, or health professional discussed with them the advantages and disadvantages, advantages only, disadvantages only, or neither advantages nor disadvantages of the PSA test among men ages 50 or above without a history of prostate cancer **A**) by age, **B**) by mortality risk quartile, **C**) by age among those screened in the past two years, **D**) by mortality risk quartile among those screened in the past two years, **E**) by age among those not screened in the past two years, and **F**) by mortality risk quartile among those not screened in the past two years

Among eligible respondents, 48.9% (95% CI 48.1%–49.8%) reported any prior SDM regarding prostate cancer screening, with 21.0% (95% 20.3%–21.6%) of respondents reporting hearing about both the advantages and disadvantages of the PSA test, 26.7% (95% CI 25.9%–27.4%) the advantages only, and 1.3% (95% CI 1.1%–1.5%) the disadvantages only. The receipt and content of SDM by age and mortality risk quartile are shown in Fig. 1. Respondents aged 70–74 were most likely to report any SDM (62.6%, 95% CI 60.5%–64.6%), with both younger respondents and older respondents reporting lower rates of SDM (Fig. 1A). The content of SDM was similar across age groups, with respondents more likely to have heard the advantages of the PSA test only, and few respondents reported hearing about the disadvantages of the PSA test only. The frequency of any SDM was lowest among those in the lowest mortality risk quartile, likely reflecting the lower age distribution in this group (Fig. 1B). Among respondents who reported being screened in the past two years, 14.5% of respondents stated that they had not previously engaged in any type of SDM. Moreover, the frequency of any SDM did not substantially differ by age or mortality risk quartiles among those recently screened (Fig. 1C, D). Among those not recently screened, 68.9% of respondents reported no prior SDM. The frequency of SDM modestly increased in older age groups and higher mortality risk quartiles among those who were not screened in the prior two years (Fig. 1E, F). The proportions of respondents who discussed the advantages and disadvantages of prostate cancer screening are shown in Supplemental Fig. 2 by age and mortality risk quartile independently and stratified by screening status.

**Fig. 2:**
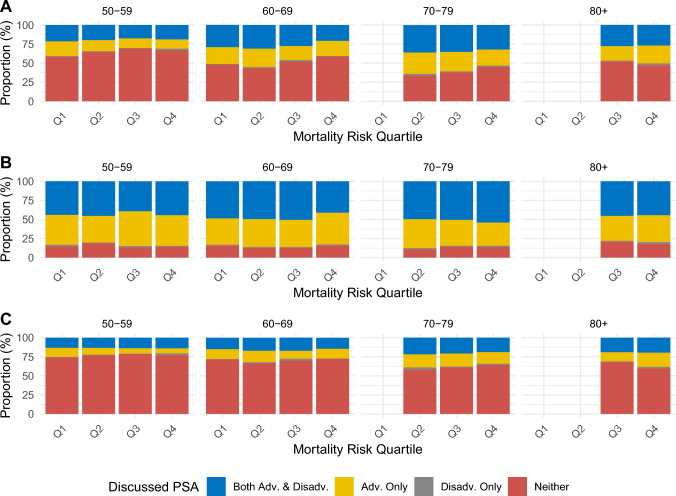
Proportions of men who report that a doctor, nurse, or health professional discussed with them the advantages and disadvantages, advantages only, disadvantages only, or neither advantages nor disadvantages of the PSA test among men aged 50 or above without a history of prostate cancer by mortality risk quartile within age group **A**) overall **B**) among those screened in the past two years, and **C**) among those not screened in the past two years. No men aged 70 or older were included in the lowest mortality risk quartile and no men aged 80 or older were included in the second mortality risk quartile

Similar patterns were observed when examining the joint association of age and life expectancy with receipt and nature of SDM (Fig. 2). Receipt of any SDM increased with age and modestly declined with increased mortality risk within age groups (Fig. 2A). The relative frequency of discussing both advantages and disadvantages, advantages only, or disadvantages only was consistent across strata of age and mortality risk quartile. These associations substantially attenuated or were not observed when stratifying by receipt of a PSA test in the prior two years (Figs. 2B, C).

In mutually adjusted logistic regression models, both age and life expectancy were associated with undergoing any SDM and undergoing complete SDM (Table 2). In the crude model, respondents aged 70–74 had 4.55-fold higher odds of undergoing any SDM (OR = 4.55, 95% CI 3.93–5.27) relative to respondents aged 50–54. Respondents in the highest mortality risk quartile were 46% less likely to undergo any SDM (OR = 0.54, 95% CI 0.47–0.62) compared to respondents in the lowest quartile. These associations attenuated upon adjustment for factors related to healthcare access and utilization (OR = 3.04, 95% CI 2.55–3.61 for aged 70–74 versus 50–54; OR = 0.84, 95% CI 0.71–0.99, mortality risk Q4 vs Q1). These associations were similar in crude and multivariable adjusted models evaluating receipt of complete SDM. Associations in relation to discussing the disadvantages of the PSA test are shown in Supplemental Table 2. The association between the continuous mortality risk score and receipt of any SDM, complete SDM, and hearing about disadvantages of the PSA test significantly differed by age (all *p*-heterogeneity < 0.001) with higher mortality risk suggestively associated with lower receipt of SDM among younger men but associated with higher receipt of SDM among men aged 80 + years (Supplemental Table 3).

**Table 2:** Mutually adjusted associations of age and mortality risk score quartile and undergoing any shared decision-making (SDM; discussing advantages and/or disadvantages), and of undergoing complete SDM (discussing both advantages and disadvantages

Characteristic	Any SDM		Complete SDM	
	Crude OR (95% CI)*	Multivariable-Adj. OR (95% CI)**	Crude OR (95% CI)*	Multivariable-Adj. OR (95% CI)**
Age				
50–54	Ref	Ref	Ref	Ref
55–59	1.55 (1.38–1.74)	1.49 (1.31–1.68)	1.55 (1.35–1.77)	1.49 (1.30–1.70)
60–64	2.05 (1.82–2.32)	1.87 (1.65–2.12)	1.73 (1.51–1.98)	1.57 (1.37–1.81)
65–69	3.08 (2.68–3.55)	2.30 (1.97–2.69)	2.26 (1.93–2.64)	1.78 (1.49–2.12)
70–74	4.55 (3.93–5.27)	3.04 (2.55–3.61)	2.81 (2.39–3.30)	2.05 (1.70–2.47)
75–79	3.68 (3.09–4.39)	2.60 (2.13–3.17)	2.27 (1.89–2.72)	1.71 (1.40–2.10)
80 +	3.35 (2.80–4.01)	2.30 (1.87–2.82)	2.55 (2.09–3.12)	1.93 (1.53–2.44)
Mortality Risk Quartile				
Q1	Ref	Ref	Ref	Ref
Q2	0.84 (0.76–0.93)	1.07 (0.96–1.19)	0.82 (0.73–0.92)	0.94 (0.84–1.06)
Q3	0.63 (0.56–0.72)	0.88 (0.76–1.02)	0.65 (0.56–0.75)	0.77 (0.66–0.90)
Q4	0.54 (0.47–0.62)	0.84 (0.71–0.99)	0.61 (0.53–0.71)	0.76 (0.64–0.90)

Cells show odds ratios (OR) and 95% confidence intervals (95% CI)

^*^Crude model includes age and mortality risk quartile

^**^Multivariable-adjusted model includes age, mortality risk quartile, race and ethnicity, health insurance status, income, educational attainment, employment status, marital status, and whether the respondent has someone they consider their personal doctor

Associations between age, mortality risk quartile, SDM, and receipt of prostate cancer screening in the prior two years are shown in Table 3. Screening rates were highest among respondents aged 70–74 (OR = 2.74, 95% CI 2.23–3.36 versus aged 50–54). Higher mortality risk was associated with lower likelihood of screening (OR = 0.61, 95% CI 0.50–0.74, Q4 vs Q1). All forms of SDM were associated with higher likelihood of screening. Respondents who reported discussing both the advantages and disadvantages of the PSA test had 10.45-fold higher odds of undergoing screening (OR = 10.45, 95% CI 9.37–11.65) versus those who reported no SDM. Discussing the advantages only (OR = 11.54, 95% CI 10.38–12.84) and disadvantages only (OR = 5.05, 95% CI 3.50–7.28) were both associated with higher odds of undergoing screening relative to no SDM. Two-year screening rates by age, mortality risk quartile, and SDM are shown in Supplemental Table 4.

**Table 3:** Mutually adjusted associations of age, mortality risk score quartile, and shared decision-making and receipt of prostate cancer screening in the prior two years

Covariate	OR (95% CI)*
Shared Decision-Making	
None	Ref
Advantages Only	11.54 (10.38–12.84)
Disadvantages Only	5.05 (3.50–7.28)
Both Advantages and Disadvantages	10.45 (9.37–11.65)
Age	
50–54	Ref
55–59	1.43 (1.23–1.66)
60–64	2.06 (1.76–2.42)
65–69	2.33 (1.93–2.81)
70–74	2.74 (2.23–3.36)
75–79	2.59 (2.01–3.33)
80 +	1.76 (1.36–2.27)
Mortality Risk Quartile	
Q1	Ref
Q2	0.91 (0.79–1.04)
Q3	0.77 (0.64–0.91)
Q4	0.61 (0.50–0.74)

Cells show odds ratios (OR) and 95% confidence intervals (95% CI)

*OR is adjusted for race and ethnicity, income, educational attainment, employment status, marital status, and having a usual source of medical care

## Discussion

Using data from a national survey, we evaluated the relationships between age, life expectancy, and receipt of SDM and prostate cancer screening among older males. Our results suggest that regardless of age group and life expectancy, SDM is an underutilized tool in the clinical setting despite national guideline recommendations. Respondents that reported any SDM were more likely to hear about only the advantages of screening, rather than complete SDM discussing both the advantages and disadvantages. When evaluating age and life expectancy jointly, SDM rates increased with age, peaking at ages 70–74, and were modestly lower among men with lower life expectancy. Receipt of any SDM, irrespective of content, was associated with a higher likelihood of recent screening.

Estimates for the frequency of SDM are highly variable, with a recent review finding prevalences ranging from 11 to 98%, and large heterogeneity in the proportions of individuals hearing about the advantages or disadvantages of screening [15]. In line with prior studies, we observed that SDM increases with age, with respondents aged 70–74 most likely to report any history of SDM and recent screening [14, 19, 20]. Prostate cancer screening rates are modestly lower among males with lower life expectancy [8, 11]. A recent analysis by Dalela et al. found that life expectancy explained < 1% of the variability in prostate cancer screening rates (< 5% among males aged 65 +) [11]. To our knowledge, life expectancy has not been previously evaluated in relation to SDM in a large national study. Our findings suggest mortality risk is inversely associated with undergoing SDM, with a similar magnitude to its association with receipt of screening. Importantly, however, the nature of SDM conversations with respect to discussing advantages and/or disadvantages of screening does not substantially differ by life expectancy. Even at older ages and among individuals with lower life expectancy, advantages-only SDM is the most common SDM type. Fewer men hear about potential harms of PCa screening even though large, randomized screening trials did not find a benefit for men ages 70 + years, and the risks of overdiagnosis are higher for these men [1–3, 21, 22].

Overall, our findings indicate that current SDM and prostate cancer screening practices do not closely follow guidelines from the USPSTF or other organizations, as SDM and PCa screening were routinely conducted in older patients and those with high mortality risk. More than half of respondents reported no prior SDM. The frequency of no prior SDM was ~ 15% among those recently screened and nearly 70% among those not recently screened, suggesting that patient preferences and values were not incorporated into decision-making regarding screening for these respondents. Moreover, in our study, any form of SDM—discussing advantages, disadvantages, or both—was strongly associated with undergoing PSA screening, and most respondents who reported any SDM were recently screened, irrespective of SDM content. In total, these findings suggest that SDM has not been used in its intended way to foster a comprehensive review of the benefits and harms associated with PSA screening, and that providers with uncertainty about the efficacy of prostate cancer screening may not be initiating SDM.

Our findings show that a significant number of respondents older than 70 years and/or with limited life expectancy still undergo SDM and PCa screening, conflicting with guideline recommendations from the USPSTF and other organizations. In fact, in our study population, those aged 70–74 underwent SDM and PCa screening at the highest rates of any age group. What frequency of SDM represents the optimal level for these groups is unclear. Given guideline recommendations against routine screening in these groups, minimal SDM should be needed as these are populations for which the harms of screening likely outweigh the benefits, making screening imprudent irrespective of patient preferences and values. However, in practice, screening remains relatively common in these populations. Accordingly, if men continue to undergo screening beyond the recommended age range and health status, they should at least engage in SDM prior to screening to ensure adequate discussion of the benefits and harms of continued screening. Our findings suggest that neither of these optima are occurring, as SDM peaks among men after age 70 and among older men receiving guideline-discordant screening; only one-third report previously engaging in complete SDM that covered the advantages and disadvantages of screening.

Multiple barriers likely exist in the implementation of effective SDM. These issues arise as primary care physicians point to lack of time as the single most common barrier to SDM [23]. Moreover, provider perspectives on the efficacy of prostate cancer screening have also been shown to influence the likelihood of SDM and may influence the content of SDM conversations [23, 24]. With this, one potential facilitator of SDM is having a single healthcare provider, as this may lead to increased trust in discussing a potentially sensitive topic [25, 26]. Decision tools and risk calculators may further help facilitate such discussions, but many decision aids for prostate cancer screening have been developed, and adoption is limited to date [15]. Future research is needed to better understand how SDM should be approached in the clinical setting and the type of toolkit that can help aid the conversation. However, given that provider perspectives may influence the content of SDM conversations, this highlights the importance of further emphasis on adequate SDM, with advantages and disadvantages of PCa screening discussed, as a way to better promote PCa screening in those likely to clinically benefit.

Our study is strengthened by the use of the BRFSS, a population-based survey generalizable to the U.S. community-dwelling population. Our study is not without limitations. First, SDM and receipt of prostate cancer screening were self-reported, raising concerns of recall bias. Prior studies have shown that self-reporting of prostate cancer screening is overestimated relative to medical records [27, 28]. Conversely, physician-reported estimates of SDM prevalence tend to be higher than patient-reported estimates, and thus, SDM may be underreported in the BRFSS [15]. Our definition of SDM included discussion of advantages and/or disadvantages of the PSA test, but the time spent on advantages and disadvantages in the SDM process is unknown and could potentially vary significantly between providers. Unlike for items regarding screening, the BRFSS items for SDM are not time period-dependent, and thus, reported SDM conversations may have occurred prior to survey response (and concurrent assessment of health status). Lastly, the life expectancy estimator has been previously validated, but there is likely measurement error in this estimate [17, 29]. However, it is unlikely that the extent of measurement error for life expectancy would differ with respect to whether a respondent underwent SDM.

In conclusion, we found in a national survey that most men aged 50 or older do not report undergoing SDM regarding prostate cancer screening, with only a subset reporting complete SDM that discusses the advantages and disadvantages of screening. Approximately, 15% of men who report undergoing recent screening do not report any prior SDM, indicating a clear target for improvement in screening practices. The receipt of SDM increases with age and decreases with mortality risk within age groups, but the content of SDM conversations does not substantially differ across age and life expectancy groups even though the benefits and harms of screening vary by these characteristics. Although age and life expectancy are sensitive topics of discussion for healthcare providers and patients, they appear to be insufficiently considered in decision-making regarding prostate cancer screening. Further work is needed to enhance and more broadly implement tools that will help target prostate cancer screening to populations most likely to benefit, such as those with lower ages and greater life expectancies.

## Supplementary Information

Below is the link to the electronic supplementary material.

Supplementary file1 (DOCX 146 KB)

## Data Availability

Data from the 2020 Behavioral Risk Factor Surveillance System are publicly available from the Centers for Disease Control and Prevention. Statistical code is available from the authors upon request.
