# Supplementary material for: Influence of life expectancy on shared decision-making for prostate cancer screening
Source: Cancer Causes Control. 2025 Sep 10;36(12):1891–900. doi: 10.1007/s10552-025-02069-1 (PMC12621089; doi:10.1007/s10552-025-02069-1)
Supplement: Supplementary file 1 — Supplementary file1 (DOCX 146 KB) [file 10552_2025_2069_MOESM1_ESM.docx]

**Supplemental Figure 1.** Flowchart for identification of analytic subpopulation in 2020 Behavioral Risk Factor Surveillance System.


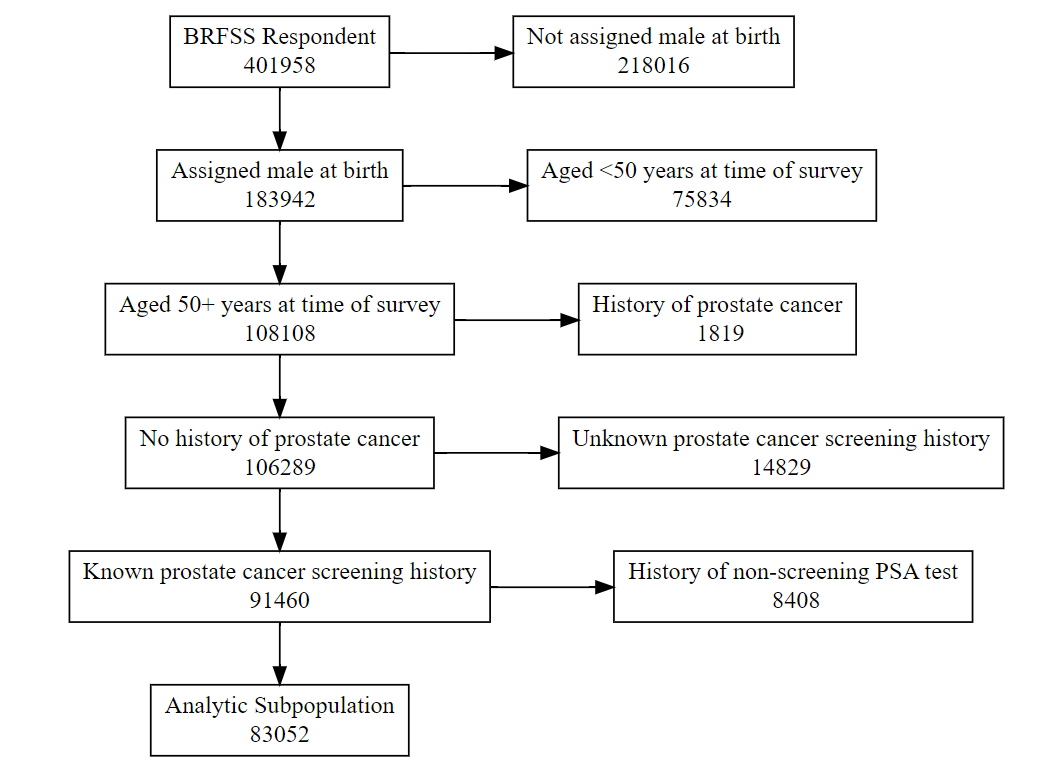


**Supplemental Table 1.** Weighted respondent characteristics by receipt of any shared decision-making (SDM) regarding prostate cancer screening.

| **Characteristic** | **Any SDM** | | **No SDM** | |
| --- | --- | --- | --- | --- |
|  | **Weighted n** | **%** | **Weighted n** | **%** |
| **Age at Survey** |  |  |  |  |
| 50-54 | 3,011,489 | 14.3 | 5,747,023 | 26.1 |
| 55-59 | 3,468,584 | 16.4 | 4,342,880 | 19.7 |
| 60-64 | 4,171,614 | 19.8 | 4,239,908 | 19.3 |
| 65-69 | 3,593,069 | 17.0 | 2,718,962 | 12.4 |
| 70-74 | 3,188,539 | 15.1 | 1,905,845 | 8.7 |
| 75-79 | 1,964,199 | 9.3 | 1,471,788 | 6.7 |
| 80+ | 1,690,059 | 8.0 | 1,583,915 | 7.2 |
| **Mortality Risk Quartile** |  |  |  |  |
| Q1 | 6,202,315 | 29.4 | 7,860,120 | 35.7 |
| Q2 | 7,443,636 | 35.3 | 6,916,361 | 31.4 |
| Q3 | 3,350,263 | 15.9 | 3,079,558 | 14.0 |
| Q4 | 4,091,339 | 19.4 | 4,154,280 | 18.9 |
| **Race and Ethnicity** |  |  |  |  |
| Hispanic/Latinx | 1,902,224 | 9.0 | 3,515,874 | 16.0 |
| Non-Hispanic American Indian/Alaska Native | 124,873 | 0.6 | 275,739 | 1.3 |
| Non-Hispanic Asian or Pacific Islander | 740,383 | 3.5 | 905,832 | 4.1 |
| Non-Hispanic Black | 2,246,409 | 10.7 | 2,264,433 | 10.3 |
| Non-Hispanic Multiracial | 144,117 | 0.7 | 227,860 | 1.0 |
| Non-Hispanic White | 15,460,971 | 73.3 | 14,240,084 | 64.7 |
| Other or Unknown | 468,574 | 2.2 | 580,498 | 2.6 |
| **Annual Household Income** |  |  |  |  |
| <$25,000 | 3,042,073 | 14.4 | 5,216,419 | 23.7 |
| <$50,000 | 3,436,956 | 16.3 | 4,193,866 | 19.1 |
| <$75,000 | 2,924,786 | 13.9 | 2,717,316 | 12.3 |
| $75,000 or more | 8,908,958 | 42.2 | 6,467,624 | 29.4 |
| Unknown | 2,774,780 | 13.2 | 3,415,096 | 15.5 |
| **Educational Attainment** |  |  |  |  |
| Less than High School | 1,726,947 | 8.2 | 4,241,907 | 19.3 |
| High School or equivalent | 4,905,198 | 23.3 | 6,762,258 | 30.7 |
| Some College | 6,378,020 | 30.2 | 5,953,639 | 27.0 |
| College Graduate | 8,024,234 | 38.1 | 4,979,684 | 22.6 |
| Other | 53,154 | 0.3 | 72,833 | 0.3 |
| **Employment Status** |  |  |  |  |
| Employed | 9,644,179 | 45.7 | 11,103,743 | 50.4 |
| Unemployed | 823,208 | 3.9 | 1,400,370 | 6.4 |
| Student/Homemaker/Unable to Work | 1,379,559 | 6.5 | 2,290,738 | 10.4 |
| Retired | 9,102,768 | 43.2 | 7,014,642 | 31.9 |
| Unknown | 137,838 | 0.7 | 200,829 | 0.9 |
| **Health Insurance Status** |  |  |  |  |
| None | 830,662 | 3.9 | 2,700,690 | 12.3 |
| Insured | 20,191,475 | 95.8 | 19,221,289 | 87.3 |
| Unknown | 65,416 | 0.3 | 88,341 | 0.4 |
| **Marital Status** |  |  |  |  |
| Married/Domestic Partner | 15,408,658 | 73.1 | 13,747,323 | 62.5 |
| Divorced/Widowed/Separated | 4,230,666 | 20.1 | 5,933,372 | 27.0 |
| Never Married | 1,385,588 | 6.6 | 2,241,630 | 10.2 |
| Unknown | 62,639 | 0.3 | 87,995 | 0.4 |
| **Has Usual Source of Healthcare** |  |  |  |  |
| Yes | 19,670,136 | 93.3 | 17,232,922 | 78.3 |
| No | 1,360,384 | 6.5 | 4,598,292 | 20.9 |
| Unknown | 57,032 | 0.3 | 179,106 | 0.8 |
| **Received Screening PSA Test in Prior Two Years** |  |  |  |  |
| Yes | 12,492,428 | 59.2 | 2,110,441 | 9.6 |
| No | 8,595,125 | 40.8 | 19,899,880 | 90.4 |

**Supplemental Figure 2**. Proportions of men who report that a doctor, nurse, or health professional discussed the advantages or disadvantages of the PSA test among men ages 50 or above without a history of prostate cancer **A**) by age, **B**) by mortality risk quartile, **C**) by age among those screened in the past two years, **D**) by mortality risk quartile among those screened in the past two years, **E**) by age among those not screened in the past two years, and **F**) by mortality risk quartile among those not screened in the past two years.


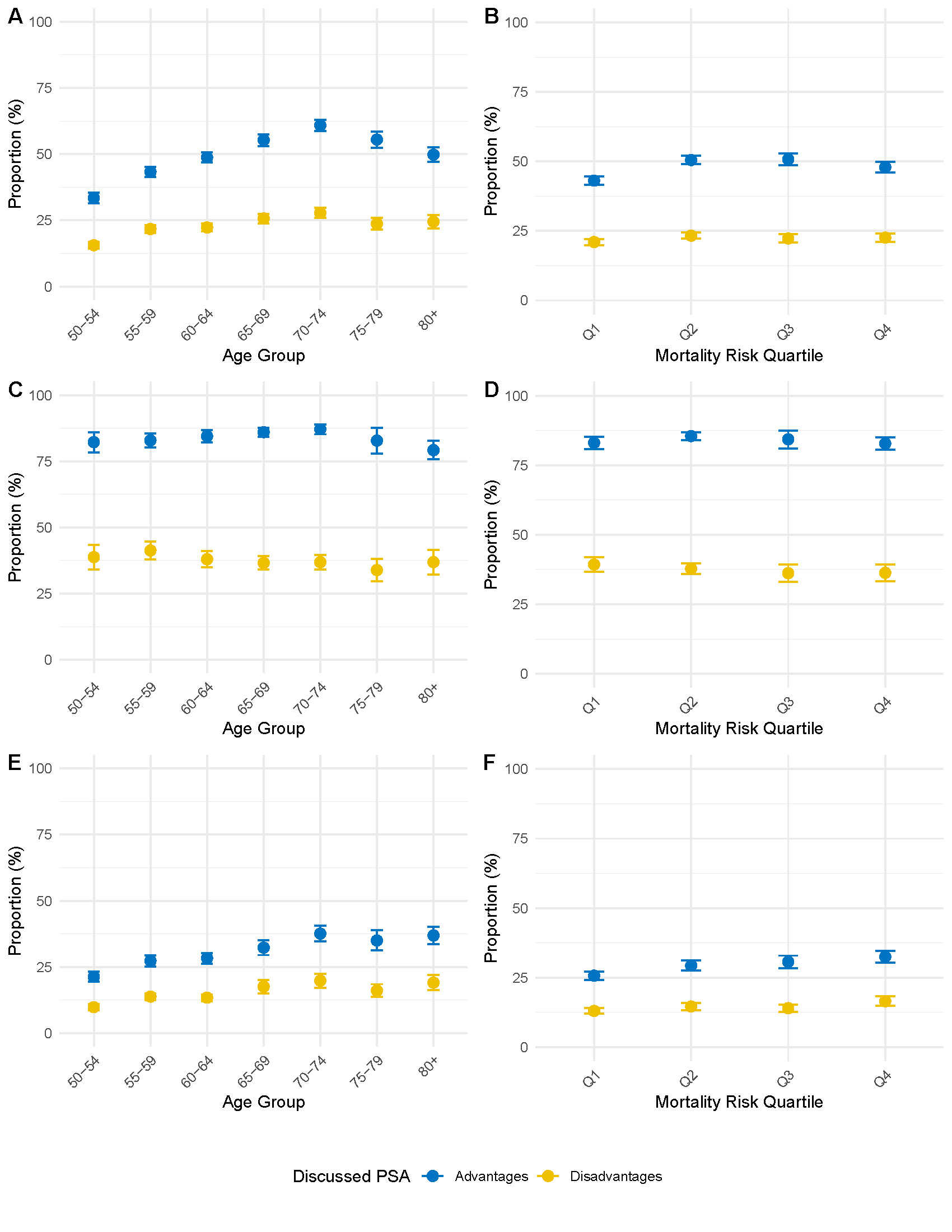


**Supplemental Table 2.** Mutually-adjusted associations of age and life expectancy and hearing about the disadvantages of prostate cancer screening. Cells show odds ratios and 95% confidence intervals.

|  | **Discussed Disadvantages** | |
| --- | --- | --- |
|  | **Crude*** | **Multivariable-Adj**** |
| **Age** |  |  |
| 50-54 | Ref. | Ref. |
| 55-59 | 1.53 (1.34-1.75) | 1.48 (1.29-1.69) |
| 60-64 | 1.69 (1.48-1.93) | 1.55 (1.35-1.77) |
| 65-69 | 2.27 (1.95-2.65) | 1.83 (1.54-2.18) |
| 70-74 | 2.80 (2.39-3.29) | 2.12 (1.74-2.58) |
| 75-79 | 2.28 (1.91-2.73) | 1.79 (1.46-2.19) |
| 80+ | 2.57 (2.11-3.13) | 2.02 (1.59-2.55) |
| **Mortality Risk Quartile** |  |  |
| Q1 | Ref. | Ref. |
| Q2 | 0.82 (0.74-0.92) | 0.93 (0.83-1.05) |
| Q3 | 0.65 (0.57-0.75) | 0.75 (0.64-0.89) |
| Q4 | 0.63 (0.54-0.73) | 0.76 (0.64-0.90) |

**Crude model includes age and mortality risk quartile*

***Multivariable-adjusted model includes age, mortality risk quartile, race and ethnicity, health insurance status, income, educational attainment, employment status, marital status, and whether the respondent has someone they consider their personal doctor*

**Supplemental Table 3.** Heterogeneity in the association between mortality risk score and receiving any shared decision-making (SDM), receiving complete SDM, and hearing about the disadvantages of prostate cancer screening. Cells show multivariable-adjusted odds ratios and 95% confidence intervals per 5-unit increase in the mortality risk score.*

| **Age** | **Any SDM** | **Complete SDM** | **Discussed Disadvantages** |
| --- | --- | --- | --- |
| 50-59 | 1.00 (0.85-1.18) | 0.78 (0.64-0.93) | 0.79 (0.65-0.95) |
| 60-69 | 0.89 (0.78-1.01) | 0.96 (0.83-1.11) | 0.94 (0.82-1.09) |
| 70-79 | 0.77 (0.65-0.91) | 0.76 (0.64-0.91) | 0.77 (0.64-0.92) |
| 80+ | 1.26 (0.99-1.61) | 1.28 (0.93-1.78) | 1.34 (0.99-1.82) |
| P-heterogeneity** | <0.001 | <0.001 | <0.001 |

**Multivariable-adjusted model includes interaction between age group and mortality risk score (continuous), and is adjusted for race and ethnicity, health insurance status, income, educational attainment, employment status, marital status, and whether the respondent has someone they consider their personal doctor*

***P-heterogeneity is from likelihood ratio test for inclusion of product terms between categorical age group and continuous mortality risk score*

**Supplemental Table 4.** Proportions of men who report receiving a screening PSA test in the past two years among men ages 50 or above without a history of prostate cancer by age, mortality risk score quartile, and shared decision-making.

|  | **% Screened (95% CI)** |
| --- | --- |
| **Shared Decision-Making** |  |
| Advantages Only | 60.8 (59.2-62.5) |
| Disadvantages Only | 36.6 (29.1-44.2) |
| Both Advantages and Disadvantages | 58.6 (57.0-60.2) |
| None | 9.6 (8.9-10.3) |
| **Age** |  |
| 50-54 | 19.8 (18.0-21.6) |
| 55-59 | 28.8 (27.1-30.5) |
| 60-64 | 36.3 (34.5-38.2) |
| 65-69 | 42.8 (40.8-44.8) |
| 70-74 | 47.1 (44.9-49.2) |
| 75-79 | 42.7 (39.6-45.9) |
| 80+ | 30.4 (27.9-32.8) |
| **Mortality Risk Quartile** |  |
| Q1 | 30.2 (28.8-31.7) |
| Q2 | 37.8 (36.4-39.1) |
| Q3 | 37.5 (35.5-39.5) |
| Q4 | 30.5 (28.8-32.3) |
